# Is Hysteroscopic Metroplasty Advisable for U2bC2V1 Malformation?

**DOI:** 10.3390/diagnostics14151649

**Published:** 2024-07-30

**Authors:** Iulian Gabriel Goidescu, Adelina Staicu, Alexandra-Andreea Poienar, Mihai Surcel, Romeo Micu, Dan Boitor Borza, Daniel Muresan

**Affiliations:** 1 Department of Obstetrics and Gynecology, “Iuliu Hatieganu” University of Medicine and Pharmacy, 400347 Cluj-Napoca, Romania; goidescu.iulian@elearn.umfcluj.ro (I.G.G.); mihai_surcel@yahoo.com (M.S.); romeo.micu@umfcluj.ro (R.M.); danboitor@yahoo.com (D.B.B.); daniel.muresan@umfcluj.ro (D.M.); 2 1st Department of Obstetrics and Gynecology, Emergency County Hospital Cluj, 400347 Cluj-Napoca, Romania; 3 Regina Maria Hospital, Endoinstitute, 400117 Cluj-Napoca, Romania; alexandra.poienar@gmail.com

**Keywords:** septate uterus, duplicated cervix, vaginal septum, hysteroscopy, metroplasty, dyspareunia, infertility, U2bC2V1

## Abstract

A complete uterine septum, with a double cervix and vaginal septum, is a complex and rare congenital genital tract anomaly. The diagnosis is difficult and often challenging, requiring complex imaging investigations and diagnostic hysteroscopy. The benefit of hysteroscopic metroplasty for this uterine malformation is still the subject of dispute. However, the potential benefits of obtaining pregnancies and reducing the rate of abortions make this surgical method a desirable one. We present a series of three cases with U2bC2V1 malformation that were diagnosed via magnetic resonance imaging (MRI), in which hysteroscopic removal of the uterine septum and resection of the longitudinal vaginal septum were performed, with the preservation of the two cervixes. All patients became pregnant after the hysteroscopic intervention and reported an improvement in dyspareunia and dysmenorrhea.

Müllerian malformations are rare anomalies that occur during the development of the paramesonephric ducts due to a failure in the fusion of these structures in the middle line.

Even though the incidence of these malformations is relatively low in the general population, their incidence increases by 7.3% in sterile women and 16.7% in women who have had recurrent miscarriages [1].

Due to the low incidence and the increased diversity of these anomalies, several classifications have been proposed, including the European Society of Human Reproduction and Embryology (ESHRE) classification [2].

A septate uterus is the result of a deficit in the reabsorption of the median septum after the fusion of the Müllerian structures. The septum can be complete or partial, but the external contour of the uterus is always normal [3]. It represents almost half of uterine malformations and is associated with recurrent miscarriage and premature birth [1].

Complete septate uterus, duplicated cervix, and longitudinal vaginal septum (U2bC2V1), a rare and complex Müllerian anomaly, with limited knowledge of its prevalence [4], is a potential cause of dyspareunia and infertility, pregnancy loss, preterm birth, and fetal growth restriction, as well as fetal malpresentation [5].

Advanced diagnostic methods can be used, such as 2D and 3D ultrasound, MRI, hysterosalpingography, foam hysterosonography, and hysteroscopy.

Two-dimensional ultrasound is a feasible investigation that is available to all clinicians; however, its diagnostic utility for identifying uterine malformations is very low. Three-dimensional ultrasound, despite requiring more advanced equipment and better examiner training, holds strong potential in obtaining better and reproducible images of the endometrial cavity [2,6]. However, the vital advantage of ultrasound lies in the possibility of a 3D transvaginal ultrasound presurgical evaluation. This technique has the potential to significantly improve hysteroscopic metroplasty [7,8,9], providing clinicians with a powerful tool for better patient care.

MRI is the gold standard and offers objective and reliable tridimensional anatomical topography. However, it has disadvantages, such as price and the requirement for qualified and experienced personnel to interpret the obtained images [2,7]. Hysterosalpingography and hysteroscopy provide valuable information about the cervix and uterine cavity; however, their diagnostic accuracy relies heavily on the examiner’s experience [2].

The necessity for surgery for a U2bC2V1 anomaly is still a topic of debate. The recommended treatment for this anomaly is through a hysteroscopic approach, which shows great potential, although no standard surgical procedure has yet been approved.

Minimally invasive hysteroscopic procedures offer clear benefits, including safety and a short recovery time, and they enable early attempts at conception.

However, they can sometimes be linked to complications such as perforation and postoperative synechia [8]; hence, they should only be considered for symptomatic patients [4], particularly those with a history of pregnancy loss.

According to the literature, abnormal blood supply to the septum leads to spontaneous abortions. Therefore, restoring the uterine cavity is a vital aspect of the hysteroscopic approach to prevent pregnancy loss [4].

This article presents a series of three cases involving patients diagnosed with U2bC2V1 anomaly, who were treated with hysteroscopic resection and obtained pregnancies in the first year after the intervention.

Case 1

A 32-year-old patient was referred for investigations about secondary infertility. She had a second-trimester miscarriage, and she complained of long-term dyspareunia.

A pelvic examination revealed a longitudinal non-obstructing vaginal septum and distinct cervices on both sides. A transvaginal 3D ultrasound showed a complete uterine septum and a duplicated cervix. The ovaries and kidneys were normal. The investigations continued with a pelvic MRI.

Hysteroscopic removal of the uterine septum up to the isthmic level, sparing the two cervices, and resection of the longitudinal vaginal septum were performed.

The resectoscope was placed into the right hemi-uterus, while a 12 Fr Foley catheter was inserted into the left hemi-uterus to provide guidance (Figure 1). The uterine septum was removed using the Collins knife, starting from the isthmic area and moving towards the uterine fundus, with the interostial plane as the limit for resection.

A single uterine cavity was obtained, as confirmed by MRI (Figure 2).

The patient was discharged on the same day with a two-month estrogen pill regimen to prevent intrauterine adhesion formation. She reported no dyspareunia after the intervention.

The hysterosalpingography performed five months after surgery confirmed the presence of a single uterine cavity with an indentation of 10 mm at the fundus and a residual fundal thickness of 18 mm (Figure 2c). No intrauterine adhesions were found.

The patient obtained pregnancy seven months following surgery with intrauterine insemination on the first attempt in a non-stimulated cycle because of the partner’s oligoasthenospermia. The pregnancy progressed without incidents, and the patient gave birth via elective cesarean section at 39 gestational weeks to a healthy male newborn weighing 3780 g.

Case 2

A 27-year-old patient was referred for dyspareunia and dysmenorrhea with no prior history of infertility.

A pelvic examination revealed a longitudinal non-obstructing vaginal septum and distinct cervices on both sides. A transvaginal 3D ultrasound showed a complete uterine septum and a duplicated cervix. The ovaries and kidneys were normal. The pre- and postoperative pelvic MRIs are depicted in Figure 3. Like in the first case, hysteroscopic removal of the uterine septum up to the isthmic level, sparing the two cervices, and resection of the longitudinal vaginal septum were performed. A single uterine cavity was obtained (Figure 2b). The patient was discharged on the same day with a two-month estrogen pill regimen to prevent the occurrence of uterine synechia.

The postoperative MRI at 2 months is depicted in Figure 2b.

The control ultrasound examination performed 5 months after surgery revealed a relatively normal single uterine cavity, and the patient was scheduled to return in another two months for hysterosalpingography (Figure 2c).

However, hysterosalpingography was not performed because the patient became pregnant spontaneously. The pregnancy proceeded without complications until 25 weeks; the patient was subsequently lost to follow-up due to a change in their country of residence.

Case 3

The third case was a 26-year-old patient who was referred for primary infertility.

A pelvic examination revealed a longitudinal non-obstructing vaginal septum and distinct cervices on both sides. A transvaginal 3D ultrasound showed a complete uterine septum and a duplicated cervix. The ovaries and kidneys were normal.

The MRI confirmed the presence of a Müllerian anomaly (Figure 4a). Like in the first two cases, hysteroscopic removal of the uterine septum up to the isthmic level, sparing the two cervices, and resection of the longitudinal vaginal septum were performed. A single uterine cavity was obtained with a slightly arcuate appearance (Figure 4b). The patient was discharged on the same day with a two-month estrogen pill regimen to prevent the occurrence of uterine synechiae.

The patient came for a check-up after three months, but due to secondary amenorrhea, the control hysterosalpingography was not performed. The patient became pregnant spontaneously, and during the pregnancy confirmation ultrasound, a decidualized region could be observed towards the right uterine horn, lateral to the area of origin of the base of the septum (Figure 4b). The pregnancy is ongoing at the time of manuscript submission and has been uncomplicated up to the third trimester of pregnancy.

The Class U2bC2V1 malformation is a rare anomaly that can be difficult to diagnose, especially when specific symptoms such as infertility and dyspareunia are absent. Advances in imaging methods such as 3D ultrasound and MRI, as well as improvements in hysteroscopic surgical instruments, have enhanced the diagnosis of this condition. These advancements have also opened possibilities for minimally invasive treatments, which are still a topic of debate in the medical community.

Despite some studies claiming that hysteroscopic metroplasty does not improve fertility and obstetric outcomes in patients with complete uterine septum, especially in asymptomatic women [8,10,11], recent meta-analyses and systematic reviews contradict this claim, particularly in cases of complex anomalies such as U2bC2V1 malformation [4,5].

Other studies have suggested that hysteroscopic metroplasty of the septum can reduce miscarriage rates and improve obstetric outcomes in patients with a history of recurrent miscarriage [12,13,14,15].

In the studies by Patton et al. [14] and Wang et al. [15], the authors found a significant decrease in miscarriage rates and preterm births following hysteroscopic intervention. This finding was further supported by a recent meta-analysis, which concluded that septum resection was linked to a lower miscarriage rate compared to untreated women. However, the analysis did not find a significant effect on live births, the clinical pregnancy rate, or preterm delivery [8].

A multicentric randomized trial published in 2021 also reported no significant difference in live birth rates and obstetric complications between patients who underwent septum resection and those who received expectant management [11].

In a recent systematic review published last year by Luca Parodi et al. [4], 336 studies published between 2000 and 2022 were reviewed. The authors identified 86 patients with U2bC2V1 malformation. Out of the 86 patients, 71 underwent hysteroscopic surgery, while 15 were treated conservatively. The authors observed that, in the surgery group, forty-seven pregnancies were achieved (forty-one live births and ongoing pregnancies and six spontaneous miscarriages), while in the non-surgery group, only eight pregnancies were achieved (seven spontaneous miscarriages and one term pregnancy). Based on these results, the authors concluded that the incidence of miscarriage was significantly lower in the surgically treated group [4].

In the three cases described in our paper, the uterine anomaly was confirmed using MRI, and post-surgical surveillance was carried out using hysterosalpingography or 3D ultrasound. Hysteroscopy is the preferred method for addressing this anomaly, distinguishing between a double cervix and a single cervix with a cervical septum, which require distinct treatment techniques.

Despite the imperfect anatomical aspect of the uterine cavity post-surgery, all three patients achieved pregnancies. Two conceived spontaneously, and one utilized intrauterine insemination due to male infertility.

It is not advisable to correct a duplicated cervix, as doing so could lead to complications such as cervical incompetence, pregnancy-related bleeding, or cervical dystocia [16]. A cesarean delivery is preferred to prevent any dystocia due to the presence of two cervices. Furthermore, patients who reported dyspareunia and dysmenorrhea experienced less pain after surgical treatment [4], and we observed the same in all three cases.

Despite the conflicting data in the medical literature about the appropriateness of hysteroscopic metroplasty in patients with U2bC2V1 malformation, the improved results, such as the reduced miscarriage rates and decreased dyspareunia and dysmenorrhea, suggest that this intervention could be reliable.

MRI or hysterosalpingography for the diagnostic and surveillance of U2bC2V1 anomaly and surgical correction via hysteroscopic metroplasty and vaginal septum section is proven beneficial for patients with a history of pregnancy loss.

## Figures and Tables

**Figure 1:**
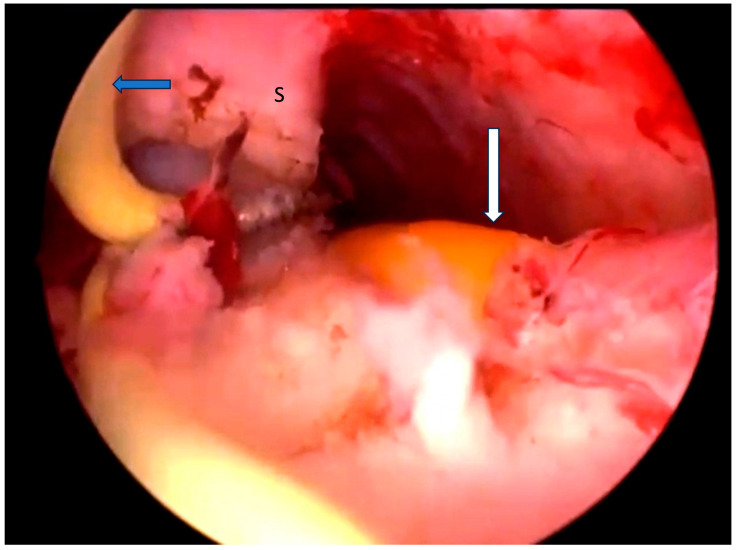
Hysteroscopic resection of the uterine septum in a patient diagnosed with U2bC2V1 anomaly using the Collins knife (blue arrow) and a Foley catheter inserted into the left hemi-uterus (white arrow); S (uterine septum).

**Figure 2:**
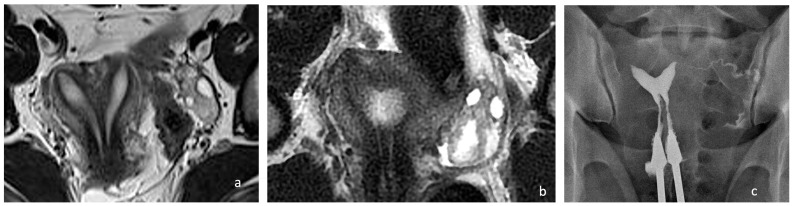
Preoperative and postoperative examination of case 1. (**a**) MRI T2, WI, confirmed the presence of a Müllerian anomaly and identified it as a complete septate uterus, duplicated cervix, and longitudinal vaginal septum (Class U2bC2V1 of the ESHRE/ESGE classification). Additionally, it provided information regarding the septum’s thickness, which measured 4–6 mm. (**b**) Postoperative MRI showing a uterine cavity. (**c**) Control hysterosalpingography performed 3 months after surgery, demonstrating a single uterine cavity.

**Figure 3:**
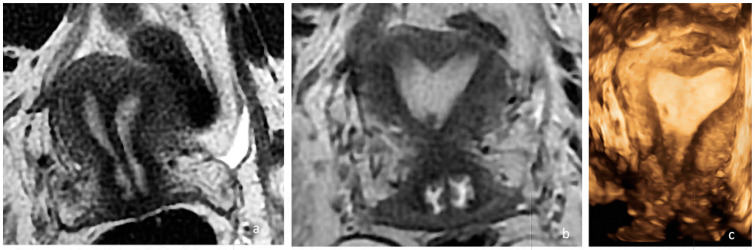
Preoperative and postoperative examination of case 2. (**a**) The preoperative MRI T2, WI, confirmed the presence of a Müllerian anomaly and identified it as a complete septate uterus, duplicated cervix, and longitudinal vaginal septum (Class U2bC2V1 of the ESHRE/ESGE classification). A complete septum with a thickness of 3 mm and a length of approximately 6 cm (measured from the intercolonial line) and 7.5 cm (measured from the level of the external contour of the uterus) was identified. (**b**) The postoperative MRI at 2 months confirmed the partial persistence of the septum in the supracervical region with the persistence of a slight indentation at the fundal level appearance of an arched uterus. (**c**) Three-dimensional ultrasonography performed 5 months postoperatively in which a normal uterine cavity was observed.

**Figure 4:**
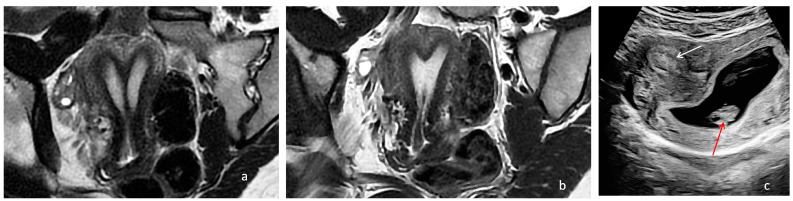
Preoperative and postoperative examination of case 3. (**a**) Preoperative MRI T2, WI, showing the two uterine cavities separated by a complete uterine septum and cervix duplication. (**b**) Postoperative MRI depicting a single uterine cavity with a slightly arcuate appearance. (**c**) Two-dimensional ultrasonography in which the embryo can be seen (red arrow) and the decidualized region is towards the right uterine horn (white arrow).
