# Correction: Goidescu et al. Is Hysteroscopic Metroplasty Advisable for U2bC2V1 Malformation? *Diagnostics* 2024, *14*, 1649

**DOI:** 10.3390/diagnostics14212396

**Published:** 2024-10-28

**Authors:** Iulian Gabriel Goidescu, Adelina Staicu, Alexandra-Andreea Poienar, Mihai Surcel, Romeo Micu, Dan Boitor Borza, Daniel Muresan

**Affiliations:** 1 Department of Obstetrics and Gynecology, “Iuliu Hatieganu” University of Medicine and Pharmacy, 400347 Cluj-Napoca, Romania; 2 1st Department of Obstetrics and Gynecology, Emergency County Hospital Cluj, 400347 Cluj-Napoca, Romania; 3 Regina Maria Hospital, Endoinstitute, 400117 Cluj-Napoca, Romania

Dan Boitor Borza was not included as an author in the original publication [1]. The corrected Author Contributions statement appears here. The authors state that the scientific conclusions are unaffected. This correction was approved by the Academic Editor. The original publication has also been updated.
